# Peak picking NMR spectral data using non-negative matrix factorization

**DOI:** 10.1186/1471-2105-15-46

**Published:** 2014-02-11

**Authors:** Suhas Tikole, Victor Jaravine, Vladimir Rogov, Volker Dötsch, Peter Güntert

**Affiliations:** 1 Institute of Biophysical Chemistry, Center for Biomolecular Magnetic Resonance, and Frankfurt Institute of Advanced Studies, Goethe University Frankfurt am Main, Max-von-Laue-Str. 9, 60438 Frankfurt am Main, Germany; 2 Graduate School of Science and Engineering, Tokyo Metropolitan University, Hachioji, Tokyo 192-0397, Japan

**Keywords:** Non-negative matrix factorization, Peak picking, NMR spectrum, Peak overlap

## Abstract

**Background:**

Simple peak-picking algorithms, such as those based on lineshape fitting, perform well when peaks are completely resolved in multidimensional NMR spectra, but often produce wrong intensities and frequencies for overlapping peak clusters. For example, NOESY-type spectra have considerable overlaps leading to significant peak-picking intensity errors, which can result in erroneous structural restraints. Precise frequencies are critical for unambiguous resonance assignments.

**Results:**

To alleviate this problem, a more sophisticated peaks decomposition algorithm, based on non-negative matrix factorization (NMF), was developed. We produce peak shapes from Fourier-transformed NMR spectra. Apart from its main goal of deriving components from spectra and producing peak lists automatically, the NMF approach can also be applied if the positions of some peaks are known a priori, e.g. from consistently referenced spectral dimensions of other experiments.

**Conclusions:**

Application of the NMF algorithm to a three-dimensional peak list of the 23 kDa bi-domain section of the RcsD protein (RcsD-ABL-HPt, residues 688-890) as well as to synthetic HSQC data shows that peaks can be picked accurately also in spectral regions with strong overlap.

## Background

The precise estimation of the frequencies of peaks in nuclear magnetic resonance (NMR) spectra is often complicated by poor signal-to-noise ratio and peak overlap. This results in only partially complete and correct peak picking. The problem aggravates especially when the peaks are highly overlapped. This is compounded by combinatorial ambiguity problems for resonance assignments and increases errors in NOE distance restraints [1]. To alleviate this problem, a more sophisticated peak decomposition algorithm, based on non-negative matrix factorization (NMF), has been developed and applied to three-dimensional (3D) NMR spectra.

Non-negative Matrix Factorization was first introduced by Paatero and Tapper as the concept of positive matrix factorization [2,3] for estimating errors in widely varying environmental data. Their work revealed the non-negativity features of the underlying data models. Lee and Seung [4,5] showed using an effective multiplicative algorithm parts-based representation of an object using NMF approach. A recent in-depth review on NMF algorithms discusses many forms of factorizations [6]. Because of the non-negativity and the sparseness constraints [7], NMF has wide applications in multidimensional data analysis [8-15]. The idea originated from the fact that in certain applications, by the rules of physics, the data quantities cannot be negative. The NMF approach was reported in application to complex metabolomic mixture analysis in two-dimensional NMR spectra [16]. Higher dimensional NMR spectral data matrices can be decomposed using NMF algorithms. The important property of NMF is the non-negative nature of the decomposed factors. Therefore, NMF processing of higher dimensional NMR spectral data can have important consequences in automated data processing.

In the automated peak picking approach peak identification is followed by the estimation of peak intensities and frequencies. Several algorithms have already been developed to perform peak picking in NMR spectra. Most of these algorithms are based on finding local maxima that fulfill certain criteria, and/or use Gaussian or Lorentzian lineshapes for lineshape fitting [17-21] by minimizing the residual squared error between the observed peak shape and the assumed lineshape. Apart from the lineshape fitting methods, PICKY [22] is another program that uses a singular value decomposition of peak components for peak picking. In general, highly overlapped peaks cause the most commonly observed problems of existing peak picking algorithms. We used the basic two-dimensional (2D) NMF model, extended sequentially to ND usage to decompose a 3D data (signal) tensor. A Euclidean distance cost function was used as a measure of factorization convergence. The approach allows applying constraints if some information is known a priori, e.g. the total number of peaks or positions in common dimensions of hyper-dimensional (HD) shapes [23]. We discuss the NMF algorithm, the conditions for unique solutions of NMF models, and its applications to decompose 3D NMR signal tensors.

## Methods

### NMF decomposition algorithm

The basic idea of spectral factorization is to represent the multidimensional NMR spectrum as well as possible by a sum of direct products of one-dimensional shapes. The latter are expected to represent the lineshapes of resonances. In this way, i.e. if each one-dimensional shape represents a resonance line, possible overlap is deconvoluted and the factorization of the spectrum is equivalent to peak picking. The exact peak positions can simply be obtained by determining the (interpolated) maxima within the one-dimensional shapes. If it is known that the real signals in a spectrum are non-negative, a better result can be expected by introducing this condition into the factorization algorithm.

The non-negative factorization (NMF) problem may be described as follows. Given the observed data matrix *Y* = [*y*(1), *y*(2), …, *y*(*T*)] ∈ *R*^*m*x*T*^ with *Y* ≥ 0. The solution is to find two matrices with only non-negative elements, the basis or mixing matrix *A* ∈ *R*^*m* × *r*^ and the source matrix *X* = [*x*(1), *x*(2), …, *x*(*T*)] ∈ *R*^*r* × *T*^, where *r* represents the number of true components [24]. The source matrix is expected to produce the unknown latent components of the original data matrix *Y*. The problem is to factorize the given data matrix such that it minimizes the squared Euclidean distance between the observed data matrix and the product of two non-negative data matrices i.e.

Y=AX+N,

with *A* ≥ 0 and *X* ≥ 0, where *N* ∈ *R*^*m*x*T*^ is a noise or error matrix that is to be minimized.

The divergence cost function is expressed in terms of the squared Euclidean distance given as

$$D_{F}\left(A,X\right)=∥Y-\mathrm{AX}{∥}^{2}$$

The objective is to minimize the divergence of this function using a standard gradient descent technique. The divergence is calculated by component-wise calculation of the distance between matrixes Y and AX. The minimization is achieved using multiplicative update rules that update the matrices *A* and *X* iteratively until a minimum squared Euclidean distance is reached. The updates may be performed until as much minimum possible distance leading to a nonnegative solution is achieved. Any increase in the squared Euclidean distance may lead to an incorrect solution.

In matrix notation, the multiplicative update rules become

$$A\leftarrow A⊙YX^{T}⊘\mathrm{AX}X^{T}$$

$$X\leftarrow X⊙A^{T}Y⊘A^{T}\mathrm{AX}$$

where ⊙ and ⊘ represent component-wise multiplication and division, respectively [25]. The proposed multiplicative update rules were originally introduced in the image space reconstruction algorithm (ISRA) [26]. The algorithm performs minimization of the squared Euclidean distance cost function using a gradient descent technique. The technique uses alternate switching between sets of parameters to generate updates on the matrices *A* and *X* until convergence is reached. The original ISRA algorithm used multiplicative updates rules by updating only the matrix *X* iteratively and assuming the matrix *A* to be known [25,26]. The convergence to a nonnegative solution is obtained for any positive starting point given that the original input matrices contain hidden source components [27].

### Extension to 3D non-negative tensor factorization

**Figure 1:**
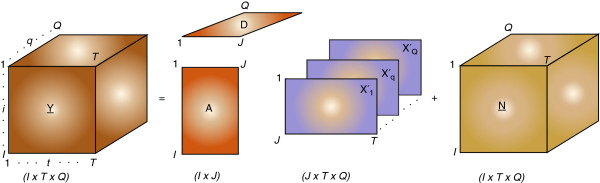
**Illustration of the NTF2 model for peak picking a 3D NMR spectrum.** The matrix *Y* is a 3D input NMR spectral data matrix. *A*_*q*_ and *X*_*q*_ are 2D matrices factorized from *q*^th^ data plane of matrix *Y* representing the basis matrix and the source component matrix, respectively. *N* is the 3D matrix representing the residual error of the non-negative matrix factorization.

The 3D non-negative tensor factorization (NTF) model may be defined as an extension of the basic 2D models. Some 3D NTF models can be solved using the basic 2D NMF models referred to as the NTF2 model [24]. The model is illustrated in Figure 1 and is described as follows.

$$Y_{q}=AD_{q}X_{q}^{'}+N_{q}=AX_{q}+N_{q},\left(q=1,\ldots ,Q\right)$$

where *Yq* = [*y*_*itq*_] ∈ *R*^*I*x*T*^ is a *q-*th frontal slice (matrix) of the observed 3D data (signal) tensor *Y* ∈ *R*^*I*x*T*x*Q*^. The component matrices *A* = [*a*_*ij*_] = [*a*_1_,*a*_2_, …, *a*_*j*_] ∈ *R*^*I*x*J*^ is a mixing or basis matrix and *X*_*q*_ ' = [*X*_*jtq*_] ∈ *R*^*J*x*T*^gives unknown normalized hidden components in *q-*th slice. The matrix *X*_*q*_ = *D*_*q*_*X*_*q*_ ' = [*X*_*jtq*_] ∈ *R*^*J*x*T*^ represents re-normalized source components and *N*_*q*_ = [*n*_*itq*_] ∈ *R*^*I*x*T*^ represents the *q*-th frontal slice of the tensor *N* ∈ *R*^*I* × *T* × *Q*^ representing noise in the input matrix *Y*.

### Determining the true number of components

For the NMF/NTF models the true number of components *r* plays an important role in reaching convergence because an approximate valid model is instrumental in capturing the true underlying structure in the data. The true number of components may be obtained using several approximate and heuristic techniques [28-32]. Different numbers of components may result in different residual minima. In the present work, we applied the following procedure for calculating the true number of components. We observed the decay of residual values for different number of components *r* = 2, 3, 4, 5, 6, and 7. The NMF iterations were stopped when the residual showed no improvement over 10 or more consecutive iterations. The true number of components was obtained as the one that showed the minimum residual value. It was also observed that using a higher number of components than the true number did not yield better minimum residual values.

### Uniqueness conditions for the 2D NMF ambiguity problem

The quadratic cost function with respect to matrices *A* and *X* may have many local minima, which leads to rotational ambiguity of the factorized matrices [33]. Therefore the alternating minimization of the squared Euclidean cost function may not result in a unique NMF solution. However, applying some preprocessing or filtering of the input matrix is sufficient to solve the NMF problem uniquely. The preprocessing involves normalization of the input matrix or normalizing the columns of the factorized matrix *A* and/or the rows of matrix *X*. We normalized each column of the matrix *A* to unit *l*_1_-norm. In addition, we normalized the input matrix *Y* to unit length at the beginning of the factorization and later used the corresponding scaling factor to obtain the original intensity values of the component peaks.

### Peak picking of 3D NMR spectral data

The 3D NMF decomposition is performed as an extended 2D NMF decomposition as described above. Each slice of 2D data, taken from a 3D spectral data matrix *Y*, occupies data in the matrix *Y*_*q*_ representing the *q*^th^ point in the third dimension. The model factorizes peak components in one-dimensional (1D) peak shapes in the source matrix *X*_*q*_. Peak positions are obtained by fitting an ideal Gaussian shape of average linewidth to the observed component by minimizing the scalar product between the Gaussian shape and the observed component. Next, the linewidth of the peak is adapted to obtain an optimal agreement. The final peak positions are obtained by performing a three-point parabolic interpolation. The final peak lists are obtained by applying a user-defined intensity threshold.

### Spectral data sets

The NTF2 model was applied using the 2D NMF algorithm to the previously measured 3D HNCO spectrum for the 23 kDa RcsD-ABL-HPt protein construct (residues 688-890) [34]. The spectrum was measured on a Bruker AVANCE spectrometer operating at ^1^H Larmor frequency of 950 MHz. The numbers of time-domain complex data points were 128 and 90 in ^13^C and ^15^N indirect dimensions respectively. Non-uniform sampling schedule was employed in both the indirect dimensions at a level of sparseness of 10 per cent, which acquired 1152 FIDs. The 203 amino acid protein gave rise to 195 expected peaks in the 3D HNCO experiment [35].

Additionally, the method was applied to a group of four synthetic signals with known positions. The synthetic signals were of different intensities and were used to construct a 2D HSQC spectrum. The efficiency of the algorithm for peak picking was assessed with different levels of noise in the spectrum. The noise was added incrementally in steps of 10 percent each. The separation of peak positions was varied from 10 to 1 points in steps of one data point each.

## Results and discussion

**Figure 2:**
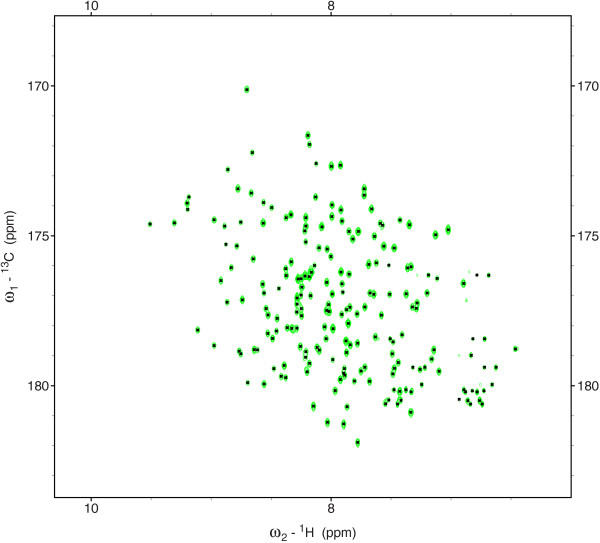
^**1**^**H-**^**13**^**C projection of the 3D HNCO NMR spectrum of RcsD-ABL-HPt (DAH) construct showing the peaks picked using the NTF2 model.** Picked peaks are marked by a cross at the peak center.

**Figure 3:**
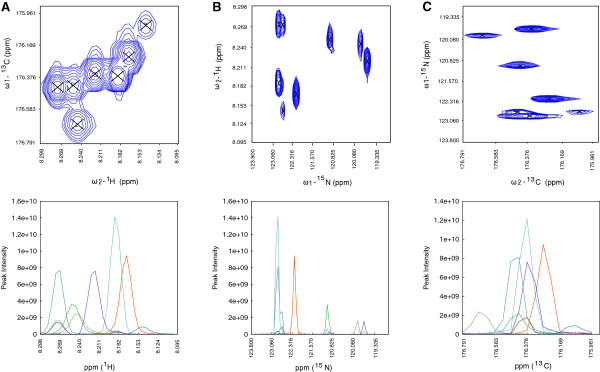
**Non-negative matrix factorization of overlapped peaks of a small region (**^**1**^**H: 8.095–8.298ppm,**^**15**^**N: 118.813–123.806ppm,**^**13**^**C: 175.910–176.791ppm) of the 3D HNCO spectrum of the RcsD-ABL-HPt (DAH) protein. A)**^1^H-^13^C projection. **B)**^1^H-^15^N projection. **C)**^13^C-^15^N projection. The upper row shows the peaks in 2D projections. The lower row shows the peaks factorized in 1D shapes from the corresponding projections. The peaks positions and intensities were obtained using a three-point parabolic interpolation.

Figure 2 shows peaks picked from the 3D HNCO spectrum of the RcsD-ABL-HPt protein on a ^1^H-^13^C projection. The model was able to pick 201 backbone and side-chain cross peaks from the HNCO spectrum. The peak list of 201 peaks is given in Additional file 1: Table S1. The NTF2 decomposition of a small region of about eight overlapped peaks of the 3D HNCO experiment is shown in Figure 3. The peak shapes determined for each dimension are plotted below each 2D projection. Peak shapes appearing in the same color in each 2D projection define one peak in the 3D spectrum. Table 1 lists the eight peaks picked in this overlap region with their assignments.

**Table 1:** **Peak list obtained by applying the NTF2 model to the overlapped region of the 3D HNCO spectrum of the RcsD-ABL-HPt construct shown in Figure**3

**Peak position (ppm)**			**Peak**	**Peak**
^**1**^**H**	^**15**^**N**	^**13**^**C**	**intensity**	**assignment**
8.275	122.868	176.380	8.013 × 10^9^	C860
8.184	122.890	176.380	1.434 × 10^10^	E861
8.271	122.861	176.420	2.011 × 10^9^	L859
8.142	122.732	176.018	9.192 × 10^8^	R824
8.241	121.000	176.412	3.430 × 10^9^	R689
8.167	122.246	176.243	9.482 × 10^9^	Q858
8.245	119.853	176.731	2.509 × 10^9^	R726
8.219	119.612	176.367	1.157 × 10^10^	T840

Peak assignments are shown in the last column.

It can be seen that the overlapped peaks were well decomposed in all three dimensions of the HNCO experiment. Among the 201 peaks picked from the HNCO spectrum there were about 23 peaks that were overlapped in one or more dimensions. The NTF2 model was able to decompose all the overlapped peaks. Table 1 shows two peaks assigned to Cysteine 860 and to Leucine 859 which are overlapped in all three dimensions. The peaks for these two residues were well decomposed in all three dimensions as shown in Figure 3. This shows that correct peak picking of 3D NMR spectral data especially in overlapped regions is possible using the NTF2 model.

**Figure 4:**
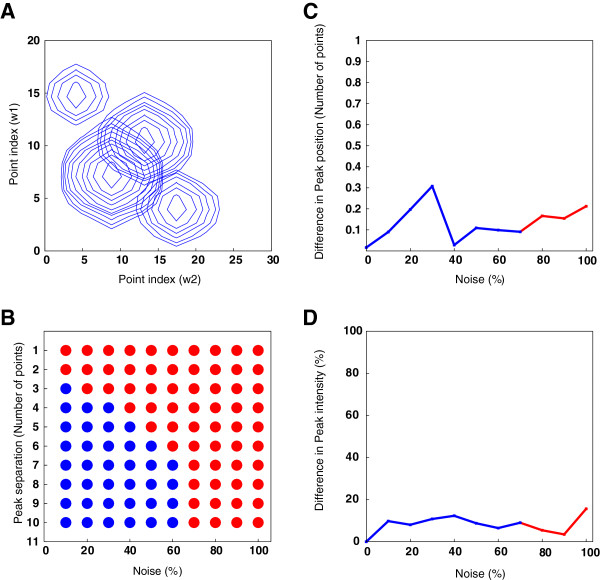
**Noise and peak overlap tolerance of the NTF2 model for peak factorization of a synthetic HSQC spectrum.A)** HSQC spectrum constructed using four synthetic signals. **B)** Effects of noise and peak overlap on peak picking. The amount of noise in the spectrum is shown on the *x*-axis. The *y*-axis shows the peak separation in number of points. Red circles indicate that peaks were incorrectly picked. Blue circles show that the peaks were correctly picked upon factorization. **C)** Effects of the amount of noise on the peak position determination. Differences in the peak position in number of points are shown on the *y*-axis. The *x*-axis shows the amount of noise in the spectrum. Blue points indicate that the peak was correctly picked. Red points indicate that the peak was obtained with incorrect parameters because of higher noise in the spectrum. **D)** Effects of the amount of noise on the peak intensity: The *x*-axis shows the amount of noise present in the spectrum. Differences in the peak intensity of the peak are shown on the *y*-axis. Blue points show that the peak was distinguishable from the noise. Red points show that the peak was obtained with incorrect parameters upon factorization.

The HSQC spectrum built using four synthetic signals is shown in Figure 4A. The results of peak picking show that the algorithm could tolerate up to 60% noise in the spectrum when the peaks were separated by at least 7 points from each other. As the peaks were moved closer to each other by one point at each step, the noise tolerance started to drop. The result is shown in Figure 4B. When peaks were closer than two points, even 10% noise in the spectrum generated incorrect peak intensities.

The change in peak position of the peak with the highest intensity with varying levels of noise in the spectrum is plotted in Figure 4C. The result shows that the algorithm could tolerate up to 70% noise in the spectrum for factorizing the peak shape at its true position. It may be noted that the known peak position is being observed for its change with increasing noise in the spectrum. Therefore, with higher than 70% of noise in the spectrum, the peak appeared at its true position albeit with incorrect linewidth and intensity. Many other peaks resembled the true peak in the factorized 1D components (Matrix *X*_*q*_). Therefore, it may be concluded that the algorithm could not reliably determine the true peak when more than 70% noise were present in the spectrum. Blue points indicate that the algorithm was able to distinguish the peak from noise. Red points indicate that many other peaks resembled the true peak because of higher noise in the spectrum. Because the original position of the peak was known, the difference in peak position could still be plotted in Figure 4C at noise levels higher than 70%. However, the matrix factorization residual and the number of peaks picked in each component were at unacceptable levels.

Similarly, the effect of noise on the change in peak intensity was observed on the peak selected for Figure 4C. Figure 4D shows the change in peak intensity with increasing level of noise in the spectrum. The algorithm could tolerate up to 70% noise in the spectrum for correct peak picking. The peak may be accepted or rejected at a different intensity depending on the user intensity tolerance threshold with higher noise levels in a spectrum. With more than 70% noise in the spectrum, incorrect intensities and linewidths were obtained after factorization.

In general, the method works well on overlapped peaks for two different reasons. First, the 3D peak picking data is reduced to a 2D peak picking data matrix. The 2D factorization is performed at all points in the third dimension. In case of overlapped peaks, for example, if two peaks are separated by only one or two points, the factorization can give two peaks separately in each data plane from the points that separate the two peaks. This becomes highly improbable in case of peak picking based on lineshape fitting. Second, if the peak position from one of the dimensions is known *a priori* then the 2D data matrix comprising unknown peak position can be factorized to get the peak position in the other dimension(s). The method has useful consequences when peaks are to be picked from hyper-dimensional data matrices as discussed below.

The usefulness of the NTF2 model becomes apparent when multi-dimensional NMR spectra have commonly referenced dimensions. Thus, if the peak positions from an already measured more sensitive NMR experiment such as 3D HNCO are known, then the same peak positions can be used to pick peaks in other spectra that have dimensions in common with HNCO, for example, ^1^H and/or ^15^N. Peak picking overlapped regions becomes easier with the NTF2 model if each common dimension has consistent spectral referencing such as the spectral width, the carrier frequency positions and the number of sampled points. Theory suggests that the components in matrix *A* are assumed known and the matrix *X* gives the hidden source components of the input matrix *Y*[25]. The advantage that the NTF2 model offers in decomposing a 3D NMR spectral data tensor is that peak positions from any *two* dimensions can be assumed known. Therefore, the reduced NTF2 model can offer accurate peak position solutions especially when the peaks are overlapped. This notion can be extended naturally to hyper-dimensional NMR spectral data tensors [23]. If peak positions from one or more dimensions are known, the peaks can be picked in the remaining dimensions by appropriately selecting the corresponding 2D data planes containing the picked peaks from the 3D data tensors.

## Conclusion

We have developed a method based on non-negative matrix factorization that can be used for peak picking 3D NMR spectral data tensors. Our results demonstrate that the method is particularly useful for picking overlapped peaks. Additionally the method can be easily extended for peak picking three- or higher-dimensional NMR spectral data tensors that have commonly referenced dimensions.

## Competing interests

The authors declare that they have no competing interests.

## Authors’ contributions

ST and VJ designed and implemented the algorithm, performed data analysis, and drafted the manuscript. VR and VD prepared protein samples and conducted the NMR experiments. PG supervised the study and drafted the manuscript. All authors read and approved the final manuscript.

## Supplementary Material

Additional file 1: Table S1 Peak list obtained by applying the NTF2 model to the entire 3D HNCO spectrum of the RcsD-ABL-HPt construct shown in Figure 2. Click here for file

## References

[B1] Güntert P Automated NMR protein structure calculation Prog Nucl Magn Reson Spectrosc 2003 43 105 125 10.1016/S0079-6565(03)00021-9

[B2] Paatero P Tapper U Positive matrix factorization - a nonnegative factor model with optimal utilization of error-estimates of data values Environmetrics 1994 5 111 126 10.1002/env.3170050203

[B3] Paatero P Least squares formulation of robust non-negative factor analysis Chemometrics Intellig Lab Syst 1997 37 23 35 10.1016/S0169-7439(96)00044-5

[B4] Lee DD Seung HS Learning the parts of objects by non-negative matrix factorization Nature 1999 401 788 791 10.1038/44565 10548103

[B5] Lee DD Seung HS Algorithms for non-negative matrix factorization Adv Neur In 2001 13 556 562

[B6] Wang YX Zhang YJ Nonnegative matrix factorization: a comprehensive review IEEE Trans Knowl Data Eng 2013 25 1336 1353

[B7] Hoyer PO Non-negative matrix factorization with sparseness constraints J Mach Learn Res 2004 5 1457 1469

[B8] Berry MW Browne M Langville AN Pauca VP Plemmons RJ Algorithms and applications for approximate nonnegative matrix factorization Comput Stat Data Anal 2007 52 155 173 10.1016/j.csda.2006.11.006

[B9] Buciu I Non-negative matrix factorization, a new tool for feature extraction: theory and applications Int J Comput Commun 2008 3 67 74

[B10] Pauca VP Piper J Plemmons RJ Nonnegative matrix factorization for spectral data analysis Linear Algebra Appl 2006 416 29 47 10.1016/j.laa.2005.06.025

[B11] Plemmons R Chung IM Nonnegative matrix factorization and applications Bull of the Int'l Linear Algebra Soc 2005 34 2 7

[B12] Cichocki A Lee H Kim YD Choi S Non-negative matrix factorization with alpha-divergence Pattern Recog Lett 2008 29 1433 1440 10.1016/j.patrec.2008.02.016

[B13] Zafeiriou S Tefas A Buciu I Pitas I Exploiting discriminant information in nonnegative matrix factorization with application to frontal face verification IEEE Trans Neural Networks 2006 17 683 695 10.1109/TNN.2006.873291 16722172

[B14] Wang W Squared Euclidean distance based convolutive non-negative matrix factorization with multiplicative learning rules for audio pattern separation 2007 Cairo, Egypt: In Proceedings of the IEEE International Symposium on Signal Processing and Information Technology: 15–18 December 2007 347 352

[B15] Kim H Park H Sparse non-negative matrix factorizations via alternating non-negativity-constrained least squares for microarray data analysis Bioinformatics 2007 23 1495 1502 10.1093/bioinformatics/btm134 17483501

[B16] Snyder DA Zhang F Robinette SL Bruschweiler-Li L Brüschweiler R Non-negative matrix factorization of two-dimensional NMR spectra: application to complex mixture analysis J Chem Phys 2008 128 052313 10.1063/1.2816782 18266430

[B17] Kleywegt GJ Boelens R Kaptein R A versatile approach toward the partially automatic recognition of cross peaks in 2D ^1^H NMR spectra J Magn Reson 1990 88 601 608

[B18] Johnson BA Blevins RA NMR View - a computer program for the visualization and analysis of NMR data J Biomol NMR 1994 4 603 614 10.1007/BF00404272 22911360

[B19] Delaglio F Grzesiek S Vuister GW Zhu G Pfeifer J Bax A NMRPipe - a multidimensional spectral processing system based on Unix pipes J Biomol NMR 1995 6 277 293 8520220 10.1007/BF00197809

[B20] Koradi R Billeter M Engeli M Güntert P Wüthrich K Automated peak picking and peak integration in macromolecular NMR spectra using AUTOPSY J Magn Reson 1998 135 288 297 10.1006/jmre.1998.1570 9878459

[B21] Goddard TD Kneller DG Sparky 3 2001 San Francisco: University of California

[B22] Alipanahi B Gao X Karakoc E Donaldson L Li M PICKY: a novel SVD-based NMR spectra peak picking method Bioinformatics 2009 25 i268 i275 10.1093/bioinformatics/btp225 19477998 PMC2687979

[B23] Jaravine VA Zhuravleva AV Permi P Ibraghimov I Orekhov VY Hyperdimensional NMR spectroscopy with nonlinear sampling J Am Chem Soc 2008 130 3927 3936 10.1021/ja077282o 18311971

[B24] Cichocki A Amari S Adaptive Blind Signal and Image Processing 2003 New York: Wiley

[B25] Byrne CL Accelerating the EMML algorithm and related iterative algorithms by rescaled block-iterative methods Ieee T Image Process 1998 7 100 109 10.1109/83.650854 18267383

[B26] Daube-Witherspoon ME Muehllehner G An iterative image space reconstruction algorithm suitable for volume ECT IEEE Trans Med Imaging 1986 5 61 66 18243988 10.1109/TMI.1986.4307748

[B27] De Pierro AR On the convergence of the iterative image space reconstruction algorithm for volume ECT IEEE Trans Med Imaging 1987 6 174 175 18230445 10.1109/TMI.1987.4307819

[B28] Bro R Kiers HAL A new efficient method for determining the number of components in PARAFAC models J Chemometrics 2003 17 274 286 10.1002/cem.801

[B29] Timmerman ME Kiers HAL Three-mode principal components analysis: choosing the numbers of components and sensitivity to local optima Br J Math Stat Psychol 2000 53 1 16 10.1348/000711000159132 10895519

[B30] Ceulemans E Kiers HAL Selecting among three-mode principal component models of different types and complexities: a numerical convex hull based method Brit J Math Stat Psy 2006 59 133 150 10.1348/000711005X64817 16709283

[B31] Da Costa JPCL Haardt M Roemer F Robust methods based on the HOVSD for estimating the model order in PARAFAC models 2008 Darmstadt, Germany: In Proceedings of SAM 2008 – The fifth IEEE Sensor Array and Multichannel Signal Processing Workshop 21–23 July 2008 510 514

[B32] Niesing J Simultaneous component and factor analysis methods for two or more groups: A comparative study 1997 Leiden, The Netherlands: DSWO Press

[B33] Cichocki A Zdunek R Phan A Amari S Nonnegative Matrix and Tensor Factorizations: Applications to Exploratory Multi-way Data Analysis and Blind Source Separation 2009 New York: Wiley

[B34] Schmöe K Rogov VV Rogova NY Löhr F Güntert P Bernhard F Dötsch V Structural insights into Rcs phosphotransfer: the newly identified RcsD-ABL domain enhances interaction with the response regulator RcsB Structure 2011 19 577 587 10.1016/j.str.2011.01.012 21481780

[B35] Schmidt E Güntert P A new algorithm for reliable and general NMR resonance assignment J Am Chem Soc 2012 134 12817 12829 10.1021/ja305091n 22794163

