# Supplementary material for: Peak picking NMR spectral data using non-negative matrix factorization
Source: BMC Bioinformatics. 2014 Feb 11;15:46. doi: 10.1186/1471-2105-15-46 (PMC3931316; doi:10.1186/1471-2105-15-46)
Supplement: Additional file 1: Table S1 — Peak list obtained by applying the NTF2 model to the entire 3D HNCO spectrum of the RcsD-ABL-HPt construct shown in Figure 2. [file 1471-2105-15-46-S1.pdf]

Supplementary Material

Table S1 Peak list obtained by applying the NTF2 model to the entire 3D HNCO spectrum of the RcsD-ABL-HPt construct shown in Figure 2.

Index	Peak position (ppm)			Peak intensity
	^{1}H	^{15}N	^{13}C	
1	7.773	121.319	181.845	2.494×10^9
2	7.899	121.386	181.305	2.511×10^9
3	7.889	121.384	181.236	2.511×10^9
4	7.341	118.833	180.873	2.610×10^9
5	7.872	108.587	180.780	1.410×10^9
6	8.149	119.222	180.653	2.329×10^9
7	6.743	113.359	180.582	2.826×10^9
8	6.855	113.333	180.580	1.997×10^8
9	7.443	113.344	180.578	4.723×10^9
10	6.745	113.364	180.578	4.659×10^9
11	7.426	113.337	180.529	1.918×10^9
12	7.542	113.390	180.529	2.026×10^9
13	7.544	113.346	180.468	2.069×10^9
14	7.435	113.344	180.468	1.441×10^9
15	6.936	113.358	180.468	4.906×10^8
16	6.842	113.362	180.468	2.385×10^9
17	6.767	113.359	180.468	4.277×10^9
18	6.823	113.027	180.217	3.462×10^8
19	7.381	112.234	180.211	1.389×10^9
20	7.333	112.626	180.183	3.457×10^9
21	6.787	112.625	180.183	2.872×10^9
22	7.427	123.181	180.167	3.069×10^9
23	7.972	117.117	180.139	1.866×10^9
24	7.470	112.193	179.943	3.669×10^8
25	7.246	112.247	179.943	9.570×10^8
26	6.879	112.205	179.943	1.430×10^9
27	6.665	112.268	179.943	1.289×10^9
28	8.554	119.457	179.921	1.921×10^9
29	8.701	125.529	179.874	8.385×10^8
30	7.675	120.616	179.835	1.867×10^9
31	7.375	120.389	179.835	9.582×10^8
32	7.916	119.452	179.776	2.449×10^9
33	8.412	121.224	179.673	1.414×10^9
34	8.376	121.224	179.673	1.274×10^9
35	7.483	122.720	179.588	1.752×10^9
36	8.202	117.460	179.520	1.152×10^9
37	7.103	105.670	179.503	1.745×10^9

38	7.882	121.020	179.493	1.985×10^9
39	7.747	120.917	179.493	1.928×10^9
40	7.727	115.500	179.472	1.948×10^9
41	6.728	111.164	179.462	8.158×10^8
42	7.259	119.660	179.440	4.770×10^8
43	7.883	119.053	179.411	1.211×10^9
44	7.715	110.933	179.373	2.273×10^9
45	7.219	111.165	179.373	6.513×10^8
46	6.632	111.167	179.373	5.583×10^8
47	7.472	115.404	179.371	2.254×10^9
48	8.389	121.631	179.312	2.397×10^9
49	7.163	118.975	179.102	1.586×10^9
50	7.997	112.504	179.074	5.218×10^8
51	8.213	117.191	179.023	7.099×10^8
52	6.837	111.782	178.977	1.413×10^9
53	8.758	120.931	178.929	8.855×10^8
54	7.488	123.082	178.924	1.857×10^9
55	7.865	112.084	178.885	2.031×10^9
56	8.213	117.191	178.865	8.874×10^8
57	8.752	122.588	178.851	1.274×10^9
58	8.771	122.589	178.841	1.368×10^9
59	8.621	118.665	178.837	1.044×10^9
60	8.102	118.142	178.808	2.631×10^9
61	8.647	119.997	178.793	2.262×10^9
62	6.475	109.772	178.759	1.254×10^9
63	8.120	115.730	178.719	2.146×10^9
64	8.256	122.561	178.684	1.927×10^9
65	8.978	122.421	178.654	1.089×10^9
66	7.870	119.902	178.500	3.636×10^9
67	7.506	113.141	178.433	1.399×10^9
68	6.822	113.026	178.433	7.617×10^8
69	6.726	113.115	178.433	1.625×10^9
70	8.484	121.075	178.429	2.198×10^8
71	8.398	121.224	178.429	2.930×10^8
72	8.484	121.075	178.420	2.004×10^9
73	7.627	116.661	178.368	1.997×10^9
74	7.407	103.796	178.298	1.370×10^9
75	8.523	117.685	178.254	1.680×10^9
76	7.989	117.444	178.254	2.486×10^8
77	8.452	123.271	178.181	1.260×10^9
78	8.046	123.160	178.181	1.318×10^9
79	8.288	110.130	178.169	1.425×10^8
80	9.113	116.733	178.143	1.259×10^9
81	7.990	119.338	178.096	4.317×10^9
82	8.316	110.130	178.089	5.082×10^9
83	8.363	121.837	178.041	5.983×10^8

84	8.058	121.940	178.041	1.904×10^{9}
85	7.851	123.625	177.924	6.097×10^{9}
86	8.046	121.320	177.915	1.519×10^{8}
87	8.447	113.475	177.757	1.817×10^{9}
88	8.028	123.160	177.673	1.534×10^{8}
89	8.521	116.274	177.647	2.272×10^{8}
90	7.905	119.452	177.626	9.058×10^{8}
91	7.778	119.375	177.626	1.161×10^{9}
92	7.578	117.929	177.611	1.813×10^{9}
93	8.042	120.314	177.552	2.090×10^{9}
94	8.022	121.536	177.536	3.585×10^{9}
95	8.022	121.814	177.501	3.560×10^{9}
96	8.286	117.595	177.454	2.563×10^{9}
97	8.260	115.561	177.441	1.480×10^{9}
98	8.248	115.561	177.441	1.625×10^{9}
99	8.536	115.024	177.437	3.301×10^{8}
100	7.289	114.478	177.426	1.572×10^{9}
101	7.715	120.563	177.383	2.348×10^{9}
102	8.027	118.781	177.294	1.808×10^{9}
103	7.839	118.606	177.259	7.284×10^{8}
104	7.283	118.693	177.259	2.081×10^{9}
105	8.870	124.576	177.230	1.131×10^{9}
106	8.745	122.589	177.147	2.120×10^{9}
107	8.268	122.868	177.092	2.247×10^{9}
108	8.284	122.868	177.092	1.376×10^{10}
109	7.641	117.523	176.969	1.187×10^{9}
110	7.510	117.738	176.969	2.300×10^{9}
111	7.373	120.389	176.954	2.289×10^{9}
112	8.170	124.053	176.944	1.448×10^{9}
113	8.252	121.000	176.926	1.588×10^{9}
114	8.000	122.712	176.894	2.369×10^{9}
115	8.555	122.397	176.773	8.831×10^{8}
116	8.418	122.547	176.773	1.724×10^{8}
117	8.245	119.853	176.731	2.509×10^{9}
118	8.565	116.895	176.637	2.082×10^{9}
119	7.903	121.020	176.621	1.695×10^{9}
120	6.900	123.406	176.610	2.551×10^{9}
121	8.041	123.160	176.570	2.865×10^{9}
122	8.921	122.658	176.512	1.477×10^{9}
123	8.530	121.075	176.450	2.070×10^{8}
124	7.118	122.498	176.443	8.835×10^{8}
125	8.271	122.861	176.420	2.011×10^{9}
126	8.241	121.000	176.412	3.430×10^{9}
127	8.275	122.868	176.380	8.013×10^{9}
128	8.184	122.890	176.380	1.434×10^{10}
129	8.219	119.612	176.367	1.157×10^{10}

130	7.189	112.316	176.335	1.108×10^9
131	6.692	112.315	176.335	9.253×10^8
132	8.167	122.246	176.243	9.482×10^9
133	8.377	121.837	176.243	2.435×10^8
134	7.911	117.906	176.233	3.398×10^9
135	8.377	119.288	176.127	1.843×10^9
136	7.362	121.623	176.097	2.246×10^9
137	8.835	113.091	176.090	1.232×10^9
138	7.334	120.158	176.060	1.712×10^9
139	8.142	122.732	176.018	9.192×10^8
140	7.683	114.020	175.983	3.408×10^9
141	8.331	119.774	175.899	2.174×10^9
142	8.645	123.171	175.725	1.455×10^9
143	8.006	122.712	175.721	1.673×10^9
144	8.044	111.855	175.483	1.783×10^9
145	8.104	118.688	175.446	1.522×10^9
146	7.472	120.776	175.439	2.923×10^9
147	8.104	118.688	175.435	1.522×10^9
148	8.037	118.781	175.435	1.775×10^9
149	7.558	116.514	175.379	3.132×10^9
150	8.791	128.412	175.377	1.331×10^9
151	8.881	124.768	175.321	7.139×10^8
152	8.213	125.094	175.240	1.106×10^9
153	7.814	115.655	175.142	2.368×10^9
154	7.635	118.680	175.055	1.866×10^9
155	7.131	123.486	175.009	3.092×10^9
156	7.769	120.917	174.898	3.471×10^9
157	8.213	123.173	174.879	1.645×10^8
158	7.026	118.201	174.838	4.106×10^9
159	8.079	112.504	174.789	6.408×10^8
160	8.082	112.504	174.786	4.021×10^9
161	8.208	109.225	174.764	4.419×10^7
162	8.081	112.504	174.743	4.002×10^9
163	8.212	109.225	174.720	1.309×10^9
164	8.892	127.280	174.719	1.091×10^9
165	7.568	124.637	174.691	1.310×10^9
166	7.348	115.586	174.675	4.117×10^9
167	9.506	132.474	174.650	9.889×10^8
168	8.561	122.397	174.620	3.139×10^9
169	9.306	122.629	174.613	1.252×10^9
170	8.569	122.397	174.608	3.129×10^9
171	8.758	127.692	174.587	9.113×10^8
172	7.903	118.561	174.556	1.549×10^9
173	7.586	118.452	174.556	1.910×10^9
174	8.977	120.854	174.514	1.489×10^9
175	8.212	123.173	174.440	3.363×10^9

176	8.373	121.631	174.440	7.583×10^8
177	7.999	117.444	174.404	2.144×10^9
178	8.334	124.443	174.342	3.779×10^9
179	7.912	119.920	174.186	2.198×10^9
180	9.196	125.786	174.168	7.274×10^8
181	7.660	115.101	174.156	2.474×10^9
182	8.491	129.232	174.113	1.160×10^9
183	7.997	128.611	174.024	1.000×10^9
184	7.997	128.611	174.024	2.274×10^9
185	8.567	115.024	173.969	1.220×10^9
186	9.200	124.899	173.964	1.931×10^9
187	8.558	122.397	173.763	1.220×10^9
188	8.132	122.270	173.763	1.890×10^9
189	9.183	118.517	173.763	9.120×10^8
190	7.719	122.479	173.701	2.579×10^9
191	8.672	124.207	173.632	1.686×10^9
192	7.719	110.933	173.492	2.218×10^9
193	8.782	123.871	173.487	1.803×10^9
194	8.865	129.937	172.854	8.886×10^8
195	8.004	117.981	172.748	2.644×10^9
196	7.918	119.054	172.713	1.318×10^9
197	7.911	119.054	172.713	2.485×10^9
198	8.127	123.925	172.657	8.509×10^8
199	8.663	128.822	172.294	1.020×10^9
200	8.195	124.361	171.730	2.005×10^9
201	8.707	118.988	170.219	1.506×10^9
